# Sociodemographic correlates of antenatal care visits in Nepal: results from Nepal Demographic and Health Survey 2016

**DOI:** 10.1186/s12884-020-03218-x

**Published:** 2020-09-05

**Authors:** Mukesh Adhikari, Binaya Chalise, Bihungum Bista, Achyut Raj Pandey, Dipak Prasad Upadhyaya

**Affiliations:** 1 Department of Health Services, Ministry of Health and Population, Kathmandu, Nepal; 2 Department of Health Policy and Management, Yale School of Public Health, Yale University, New Haven, CT USA; 3 Graduate School for International Development and Cooperation, Hiroshima University, Hiroshima, Japan; 4 Nepal Health Research Council, Kathmandu, Nepal; 5 Nepal Health Sector Program 3/Monitoring Evaluation and Operational Research, Abt Associates, Kathmandu, Nepal; 6 Central Department of Public Health, Institute of Medicine, Tribhuvan University, Kathmandu, Nepal

**Keywords:** Pregnancy, antenatal care, quality, Nepal

## Abstract

**Background:**

Good quality antenatal care visits are crucial to reduce maternal mortality and improve overall maternal and neonatal health outcomes. A previous study on antenatal care visits analyzed the nationally representative data of 2011; however, no studies have been conducted recently in Nepal. Therefore, we analyzed the sociodemographic correlates of the frequency and quality of antenatal care among Nepalese women from the nationally representative data of 2016.

**Methods:**

We analyzed data obtained from the Nepal Demography Health Survey (2016) on antenatal care for 2761 women who had one or more births in the past three years. Our study defined ‘good quality antenatal care’ as at least a 75% score on a composite metric which was obtained by adding the weighted scores assigned to the twelve recommended components of antenatal care. We analyzed the factors associated with the frequency and quality of antenatal care by using multiple Poisson regression and multiple logistic regression.

**Results:**

While 70% of the Nepalese women surveyed had at least four antenatal care visits, only 21% of these women received good-quality antenatal care. We found that the educated women (APR: 1.12; CI: 1.05–1.19) and the women of rich wealth index (APR: 1.27; CI: 1.18–1.37) were more likely to receive a higher number of antenatal visits. In contrast, women living in rural areas (APR: 0.92; CI: 0.87–0.98), and those who had more than two children (APR: 0.88; CI: 0.83–0.93) were less likely to receive a higher number of antenatal visits. Regarding the quality of antenatal care, educated women (AOR: 1.51; CI: 1.09–2.08), women who had educated husbands (AOR: 2.11; CI: 1.38–3.22), women of rich wealth index (AOR: 1.58; CI: 1.13–2.20) and women who had intended pregnancy (APR: 1.69; CI: 1.23–2.34), were more likely to receive good-quality antenatal care.

**Conclusions:**

Observing a wide variation in the coverage of different components of antenatal care, concerned stakeholders could tailor the interventions by focusing on components with lower use. Because we found an association of myriad sociodemographic factors with the frequency and quality of antenatal care, targeted interventions are necessary.

## Background

Although the world observed significant improvement in maternal health in the last two decades, recent estimates suggest that about 808 mothers continued to die per day due to complications associated with pregnancy and childbirth [1]. More importantly, the maternal mortality ratio is highly disproportionate; about 94% of all maternal deaths in the world occur in low-income settings, and these deaths are mostly preventable [2]. Therefore, avoiding maternal deaths and promoting women’s health and well-being is essential. To achieve this, a continuum of care is necessary, for which Antenatal Care (ANC) is a core intervention [3].

ANC not only detects early risks and complications during pregnancy but it also provides health education, diagnostic tests, vaccines, and treatments [4]. As evidenced by systematic reviews and meta-analyses, ANC encourages pregnant women to deliver at the health facility, reduces maternal mortality [5], and improves neonatal outcomes [6]. Further, it also promotes a positive pregnancy experience in which every woman maintains a healthy pregnancy along with physical and sociocultural normality, self-esteem, competence, and autonomy [7].

ANC should be standardized both in terms of quantity and quality because adequate and good quality ANC improves pregnancy and neonatal health outcomes. Quantitatively, the World Health Organization (WHO) recently recommended an ANC model with a minimum of eight contacts with health workers, as evidence suggests that this new model had better outcomes in reducing perinatal mortality and improve women’s experience as compared to the older four-visit focused ANC (FANC) [7]. Qualitatively, the WHO defines “good quality” health care as care that is safe, effective, timely, efficient, equitable, and people-centered [8]. Regarding ANC, the WHO recently formulated a guideline that includes 49 ANC-related recommendations in five categories of interventions: nutrition interventions, maternal and fetal assessments, preventive measures, interventions for common physical symptoms, and a broader level of health system interventions to increase utilization and enhance the quality of ANC [7]. Some of these recommendations are general, while others are context-specific.

In Nepal, the coverage of four ANC visits is not high (69%) [9]. Further, less than 25% of women had received good-quality ANC service [10]. Although the quality of care is one of the strategic principles of the Nepal Health Sector Strategy 2015–2020 [11], further analysis of the 2015 National Health Facility Survey demonstrated that the quality of ANC services in the majority of the dimensions such as effectiveness, efficiency, and safety was poor [12]. However, the factors associated with the poor quality of ANC were not analyzed.

A prior study on utilization and quality of ANC visits was conducted by analyzing data of Nepal Demographic Health Survey 2011 [10]; however, to the best of our knowledge, we have not observed any studies in this topic in the last five years in Nepal consisting a nationally representative sample. Further, in line with the recent recommendations from the WHO to revise the ANC guidelines, it is imperative to study the factors associated with four or more ANC visits and ANC quality to devise policy and programs related to ANC services. Therefore, this study aims to investigate the socioeconomic and demographic correlates of the number and quality of ANC visits received by Nepalese women.

## Methods

### Data source

We used the Nepal Demographic and Health Survey (NDHS) 2016 with publicly available microdata from the Demographic and Health Service program to extract data from individual women about their antenatal care practices. NDHS 2016 was a nationally representative cross-sectional survey of 11,040 households selected from 383 primary sampling units through stratified multi-stage cluster sampling [9]. The survey interviewed 12,862 women ages 15–49 years out of 13,089 eligible women with a response rate of 98.3%. The survey adopted a universally standardized DHS questionnaire to collect information from the selected women about their pregnancies and birth history. The final report of NDHS 2016 elaborated a detailed description of the survey methods and sampling procedure [9]. We restricted our analyses to 2761 women who had one or more births in the three years preceding the survey. NDHS 2016 inherently asked about the history of births in the past three years of the survey. Because our study focused on the analyses of antenatal care related to live births, we excluded women who had no history of live births. Further, to minimize recall bias, we restricted our analyses on the frequency and quality of ANC visits that the women received during their most recent birth. That means if a woman had two or more births, we included the detail of ANC visits of the most recent birth only.

### Study variables

Our first outcome variable is the number of ANC visits undertaken by women during their most recent pregnancy three years preceding the survey that resulted into live births. The NDHS 2016 collected information on the number of ANC visits as well as the timing and providers of the visits. We treated the number of ANC visits, irrespective of the timing and provider, as a count variable for multivariable analysis. The quality of ANC visits is our second outcome variable. NDHS 2016 collected the information on the health service content offered to women during their ANC visits. These service components include: iron supplementation for at least 180 days, treatment for intestinal parasites, blood and urine sample test, blood pressure measurement, two or more doses of tetanus toxoid vaccine, voluntary counseling of HIV, and health education on birth preparedness.

We used the content of the ANC as well as information about the number, timing, and provider of the ANC visits to construct a composite index. We used twelve yes/no components of ANC and weighted them as follows. We assigned a score of ‘3’ to two very important components of ANC. The first includes receiving the first ANC visit in the first trimester, which important for early commencement of health education, early documentation of baseline physiological parameters, and early detection of anomalies and modifiable pre-existing conditions [13, 14]; and it also signals higher probability of continuum of care. The second includes receiving at least four ANC visits by skilled providers, which is vital for regular monitoring of physiological and laboratory parameters along with better compliance to recommended care and a higher chance of having institutional delivery [15] and better birth outcomes [13]. This component also ensures a continuum of care. Further, we assigned a score of ‘2’ to the medicinal supplements, treatments, and laboratory or physiological measurements. We assigned less weight to these components than the previous two components because they do not completely represent continuum of care; NDHS assigned ‘yes’ to these components if women received these services for at least once. Although iron intake for at least 180 days signals continuum of care, according to the Nepalese government guideline, Female Community Health Volunteers (i.e. FCHV – or health cadres classified as unskilled in terms of antenatal care) can refill iron tablets [16]. Therefore, women who received iron from FCHV will not receive skilled antenatal care. Additionally, we assigned a score of ‘1’ to the health education or counseling related components because it is not certain that having knowledge about antenatal care means that pregnant women would translate it into action. After calculating the total score, we computed the percentile rank for each woman where we considered ‘good quality ANC’ to be achieved when the total score obtained by a woman was at 75th percentile or above [17–19]. A detailed description of these items is depicted in Table 1.

**Table 1:** Components of Antenatal Care and their weighted score

Item No	Item Description	Score weight
1	Initiated first ANC within four months of pregnancy	3 = Yes, 0 = No
2	Underwent at least four ANC by skilled providers (doctor, nurse or midwife)	3 = Yes, 0 = No
3	Taken iron tablets for at least 180 days during pregnancy	2 = Yes, 0 = No
4	Received drug for treatment of intestinal parasite	2 = Yes, 0 = No
5	Blood sample tested during pregnancy	2 = Yes, 0 = No
6	Urine Sample tested during pregnancy	2 = Yes, 0 = No
7	Blood pressure measured during pregnancy	2 = Yes, 0 = No
8	Received two doses of tetanus toxoid	2 = Yes, 0 = No
9	Received counseling for voluntary test of HIV	1 = Yes, 0 = No
10	Advised for complication during pregnancy	1 = Yes, 0 = No
11	Advised where to go for pregnancy complication	1 = Yes, 0 = No
12	Advised for institutional delivery	1 = Yes, 0 = No

The independent variables in our analyses included sociodemographic factors (family size, women’s education, husband’s education, ethnicity, religion, wealth index, women’s access to media and women’s autonomy of health care), contextual factors (place of residence and ecological region) and maternal factors (intended pregnancy, history of terminated pregnancy and number of living children). Ethnicity was broadly categorized into advantaged and disadvantaged groups based on the socio-cultural hierarchy of ethnic groups in the Nepalese context. We classified Brahmin, Chhetri, and Newar ethnic groups as ‘advantaged groups’ and Dalit, Janajati, Muslim, other Terai castes and castes of ‘other’ group as ‘disadvantaged groups’ as suggested by the previous studies [20, 21]. Religion was broadly dichotomized into Hindus and non-Hindus. Educational attainment was divided into illiterate and literate where illiterate means having no education and literate means those who had primary or higher levels of schooling. Women were said to have access to the media if they read a newspaper, listened to the radio, or watched television at least once a week. Although a previous study defined women’s autonomy as her decision-making autonomy for her health care, large household purchases, and visits to family or relatives [22], we only considered women’s autonomy in terms of health care decisions. Women were said to have autonomy in health care if they responded that they usually make decisions about their health care themselves without the involvement of husband, partner, or someone else.

### Data analyses

We performed all analyses using STATA 15.1 version [23] with the survey (*svy*) set command [24]. We weighted all estimates by sample weights and presented with 95% confidence intervals (CI). For bivariate analyses to test associations between covariates and dependent variables, we performed Chi-square tests. Furthermore, considering the number of ANC as a count data, we used multiple Poisson regression with a robust variance to calculate the adjusted prevalence ratio (APR) for determinants of the number of ANC visits. To determine the association between good-quality ANC and independent variables, we computed the adjusted odds ratio (AOR) using multivariable logistic regression.

### Ethical considerations

The NDHS 2016 sought ethical approval from the Ethical Review Board (ERB) of the Nepal Health Research Council (NHRC), Nepal, and ICF Macro Institutional Review Board, Maryland, USA. Further, this survey obtained a written informed consent from each participant prior to enrollment.

## Results

We included 2761 women in the analysis who had at least one birth in the past three years preceding the survey. For both, the number and quality of ANC, we considered antenatal care received by the women in their last pregnancy.

### Number of ANC visits

More than two thirds of women (70.4%, CI: 67.4–73.2%) had four or more ANC visits (Table 2). Three variables were positively associated with the number of ANC visits in multivariable Poisson regression: the educational status of women, the educational status of the husband, women’s accessibility to media, and the wealth index of the women’s household. Literate women were expected to have 1.12 times as many ANC visits compared to illiterate women (APR: 1.12, CI: 1.05–1.19). Further, those women who had literate husbands were more likely to have a greater number of ANC visits compared to those who had an illiterate husband (APR: 1.09, CI: 1.02–1.17). Women who had access to media were expected to have 1.10 times as many ANC visits compared to those who had no access to media (APR: 1.05; CI: 1.00-1.10). As the wealth of the women’s households increased, the women were expected to have a greater number of ANC visits. Women of rich wealth index (APR: 1.27, CI: 1.18–1.37) and middle wealth index (APR: 1.16, CI: 1.09–1.23) were expected to have a greater number of ANC visits compared to women who were categorized under poor wealth index.

**Table 2:** Correlates of number of antenatal care visits for Nepalese women

	Number of **women**	≥ 4 ANC Visits						
		**Number of women**	**Percent**	**95%CI**	**PR**	**95%CI**	**APR**	**95%CI**
**Family size**								
<= 4	758	575	76.31	(72.69, 79.59)				
> 4	2003	1388	68.15	(64.67, 71.44)	0.96	(0.90, 1.01)	1.04	(0.98, 1.10)
**Place of residence**								
Urban	1581	1201	75.56	(71.70, 79.04)				
Rural	1180	762	64.25	(59.28, 68.93)	0.83	(0.78, 0.89)	0.92	(0.87, 0.98)
**Ecological region**								
Mountain	226	149	68.44	(58.69, 76.79)				
Hill	1184	889	76.63	(72.70, 80.16)	1.22	(1.09, 1.37)	1.08	(0.98, 1.18)
Terai	1351	925	66.14	(61.45, 70.53)	1.12	(1.01, 1.25)	1.01	(0.92, 1.12)
**Education status of women**								
Illiterate	792	425	52.51	(46.84, 58.11)				
Literate	1969	1538	77.94	(75.17, 80.48)	1.33	(1.24, 1.43)	1.12	(1.05, 1.19)
**Education status of husband**								
Illiterate	337	168	48.72	(41.95, 55.55)				
Literate	2412	1787	73.72	(70.90, 76.36)	1.34	(1.24, 1.45)	1.09	(1.02, 1.17)
**Ethnicity**								
Advantaged	962	769	82.70	(78.86, 85.97)				
Disadvantaged	1799	1194	64.94	(61.14, 68.56)	0.83	(0.77, 0.89)	0.89	(0.84, 0.95)
**Religion**								
Hindus	2396	1733	71.60	(68.42, 74.59)				
Non-Hindus	365	230	63.39	(55.16, 70.91)	0.93	(0.85, 1.02)	0.98	(0.91, 1.06)
**Wealth index of women’s household**								
Poor	1310	849	63.90	(59.81, 67.80)				
Middle	569	398	67.69	(62.05, 72.86)	1.14	(1.07, 1.21)	1.16	(1.09, 1.23)
Rich	882	716	79.78	(75.89, 83.18)	1.38	(1.29, 1.48)	1.27	(1.18, 1.37)
**Women’s autonomy of health Care**								
No	2210	1554	69.19	(65.73, 72.44)				
Yes	539	401	75.72	(70.62, 80.18)	1.05	(0.93, 1.19)	1.01	(0.91, 1.12)
**Women’s access to media**								
No	1289	786	59.78	(55.42, 63.99)				
Yes	1472	1177	79.12	(76.35, 81.64)	1.26	(1.20, 1.32)	1.05	(1.00, 1.10)
**Intended pregnancy**								
No	576	359	61.21	(56.02, 66.16)				
Yes	2185	1604	72.79	(69.55, 75.80)	1.08	(0.97, 1.21)	1.06	(0.96, 1.15)
**History of terminated pregnancy**								
No	2171	1556	71.08	(67.82, 74.14)				
Yes	590	407	67.82	(62.33, 72.87)	0.98	(0.90, 1.07)	1.00	(0.94, 1.08)
**Number of living children**								
Two or less	2009	1543	76.23	(73.40, 78.84)				
More than two	752	420	54.83	(49.84, 59.71)	0.77	(0.72, 0.81)	0.88	(0.83, 0.93)
**Total**	**2761**	**1963**	**70.40**	**(67.39, 73.24)**				

*PR* Prevalence Ratio, *APR *adjusted Prevalence Ratio

In contrast, women having the following sociodemographic characteristics had a higher chance of having a smaller number of ANC visits: women of rural residence, women of disadvantaged ethnic group, and those who had at least one living child (Table 2). Women residing in rural areas had only 92% as many ANC visits as women in the urban areas did (APR: 0.92, CI: 0.87–0.98). Women of the disadvantaged ethnic groups had a fewer ANC visits compared to those in advantaged ethnic groups (APR 0.89, 95% CI: 0.84–0.95). The proportion of the number of ANC visits among the women who had more than two living children was 0.88 times as many as those who had at least two living children (APR 0.88, CI: 0.83–0.93).

We did not observe any significant difference in the number of ANC visits on the basis of sociodemographic variables such as family size, religion, women’s autonomy of health care, intended pregnancy, and previous history of terminated pregnancy. Although the ecological region where the women lived was statistically significant in the univariate model, it was not significant in the multivariable model.

### Components and quality of ANC

During the last pregnancy, there was a wide variation in the proportion of women who received various components of ANC. Some components were widely utilized by women. For example, more than 9 in 10 women received two doses of tetanus toxoid during the ANC visit (93.7%; CI: 92.4–94.7%). Similarly, blood pressure measurement is another component of ANC that was commonly received by women during their ANC visits (91.8%, CI: 90.3–93.1%). The majority of pregnant women (85.8%, CI: 83.4–87.9%) received the first ANC within four months of pregnancy. In contrast, some components of ANC were less commonly received. For example, about one in ten women received counseling for a voluntary HIV test (11.0%; 95% CI: 9.6–12.5%). Likewise, only 40.5% (95% CI: 38.0-43.1%) of pregnant women had taken iron tablets for at least 180 days. The coverage of other ANC components received by women during their last pregnancy is described in Table 3.

**Table 3:** Component of antenatal care received during the last pregnancy for Nepalese women

Components of ANC	Number of women	Percent	95%CI
**Initiated first ANC within 4 months of pregnancy**			
No	339	14.17	(12.08, 16.57)
Yes	2293	85.83	(83.43, 87.92)
**Underwent at least 4 ANC by skilled providers**			
No	967	36.41	(33.43, 39.49)
Yes	1794	63.59	(60.51, 66.57)
**Taken iron tablets for at least 180 days**			
No	1583	59.47	(56.89, 62.00)
Yes	1178	40.53	(38.00, 43.11)
**Received drug for intestinal parasite**			
No	652	27.22	(24.49, 30.14)
Yes	2081	72.78	(69.86, 75.51)
**Blood sample tested during pregnancy**			
No	854	31.45	(28.54, 34.52)
Yes	1778	68.55	(65.48, 71.46)
**Urine sample tested during pregnancy**			
No	620	22.83	(20.42, 25.42)
Yes	2012	77.17	(74.58, 79.58)
**Blood pressure measured during pregnancy**			
No	226	8.21	(6.90, 9.74)
Yes	2406	91.79	(90.25, 93.10)
**Received 2 doses of tetanus toxoid**			
No	194	6.34	(5.28, 7.57)
Yes	2567	93.66	(92.43, 94.71)
**Received counseling and testing for HIV**			
No	2418	89.03	(87.46, 90.43)
Yes	342	10.97	(9.56, 12.54)
**Advised for complication during pregnancy**			
No	497	20.79	(18.23, 23.60)
Yes	2132	79.21	(76.40, 81.77)
**Advised where to go for pregnancy complication**			
No	495	20.25	(17.94, 22.78)
Yes	2134	79.75	(77.22, 82.06)
**Advised for institutional delivery**			
No	453	19.67	(17.20, 22.40)
Yes	2179	80.33	(77.60, 82.80)

About one in five pregnant women (21.5%; CI: 19.2–23.9%) received good-quality ANC (Table 4). We found ethnicity as the strongest predictor of good-quality ANC. Compared to women of advantaged ethnic groups, those of disadvantaged ethnic groups had 40% lower odds of receiving good-quality ANC (AOR: 0.60; CI: 0.46–0.78). Similarly, women who had at least two living children had 35% lower odds of receiving good-quality ANC compared to those women who had more than two children (AOR: 0.65; CI: 0.49–0.86). Women who had a literate husband were more likely to receive good-quality ANC compared to those who had an illiterate husband (AOR: 2.11; CI: 1.38–3.22). In addition, the odds of receiving good-quality ANC was as high as 1.51 times for literate women as compared to illiterate women (AOR: 1.51; CI: 1.09–2.08). Women in the rich wealth index had 1.58 times greater odds of receiving good-quality ANC compared to women in the poor wealth index (AOR: 1.58; CI: 1.13–1.93). Women who had access to media had 36% higher odds of receiving good quality ANC compared to those who had not (AOR: 1.36; CI: 1.06–1.74). When the pregnancy was intended, the women had 69% higher odds of receiving good-quality ANC compared to the women whose pregnancy was unintended.

**Table 4:** Correlates of quality of antenatal care services in Nepal

Variables	Number of **Women**	Good Quality ANC						
		**Count**	**Percent**	**95%CI**	**OR**	**95%CI**	**AOR**	**95%CI**
**Family size**								
<= 4	758	207	25.38	(21.69, 29.45)				
> 4	2003	440	19.96	(17.65, 22.50)	0.73	(0.59, 0.91)	0.98	(0.78, 1.23)
**Place of residence**								
Urban	1581	418	24.20	(20.71, 28.08)				
Rural	1180	229	18.17	(15.51, 21.19)	0.70	(0.52, 0.92)	0.94	(0.72, 1.23)
**Ecological region**								
Mountain	226	49	21.20	(15.56, 28.21)				
Hill	1184	321	25.35	(21.30, 29.87)	1.26	(0.81, 1.98)	0.86	(0.57, 1.32)
Terai	1351	277	18.67	(15.86, 21.85)	0.85	(0.56, 1.31)	0.79	(0.51, 1.23)
**Education status of Women**								
Illiterate	792	96	10.95	(8.55, 13.93)				
Literate	1969	551	25.88	(23.16, 28.79)	2.84	(2.10, 3.83)	1.51	(1.09, 2.08)
**Education status of Husband**								
Illiterate	337	27	7.22	(4.87, 10.56)				
Literate	2412	619	23.70	(21.35, 26.22)	3.99	(2.67, 5.97)	2.11	(1.38, 3.22)
**Ethnicity**								
Advantaged	962	314	31.34	(27.10, 35.92)				
Disadvantaged	1799	333	17.06	(14.77, 19.63)	0.45	(0.35, 0.58)	0.60	(0.46, 0.78)
**Religion**								
Hindus	2396	589	22.42	(20.17, 24.84)				
Non-Hindus	365	58	15.86	(10.79, 22.70)	0.65	(0.42, 1.01)	0.86	(0.57, 1.29)
**Wealth index of women’s household**								
Poor	1310	235	16.37	(14.11, 18.92)				
Middle	569	124	19.97	(16.34, 24.18)	1.27	(0.95, 1.70)	1.41	(1.03, 1.93)
Rich	882	288	28.39	(23.88, 33.38)	2.03	(1.53, 2.67)	1.58	(1.13, 2.20)
**Women autonomy in health care**								
No	2210	491	20.19	(17.88, 22.73)				
Yes	539	155	27.35	(22.47, 32.83)	1.49	(1.13, 1.97)	1.33	(0.99, 1.80)
**Women’s access to Media**								
No	1289	203	14.57	(12.29, 17.19)				
Yes	1472	444	27.11	(23.89, 30.58)	2.18	(1.72, 2.77)	1.36	(1.06, 1.74)
**Intended pregnancy**								
No	576	104	14.92	(11.61, 18.97)				
Yes	2185	543	23.15	(20.80, 25.69)	1.72	(1.30, 2.27)	1.69	(1.23, 2.34)
**History of terminated pregnancy**								
No	2171	521	22.09	(19.64, 24.75)				
Yes	590	126	19.04	(15.63, 23.00)	0.83	(0.64, 1.07)	2.34	(0.67, 1.21)
**Number of living children**								
No	2009	549	25.19	(22.48, 28.10)				
Yes	752	98	11.48	(9.235, 14.18)	0.39	(0.30, 0.50)	0.65	(0.49, 0.86)
**Total**	**2761**	**647**	**21.45**	**(19.22, 23.86)**				

*OR* Odds Ratio, *AOR *Adjusted Odds Ratio, *CI* Confidence Interval

Although women’s autonomy in health care, their place of residence, and the size of the family were statistically significant with quality ANC in the univariate model, they were not significant in the multivariable model.

## Discussion

We found that about 70% of Nepalese women with a birth history in the past three years had four or more ANC visits; however, only 21.5% of the them received good-quality ANC. Further, our study demonstrated that the number of ANC visits and good quality ANC overall were associated with myriad factors ranging from socioeconomic factors, such as the educational status of the women and their husband, to their obstetrics characteristics, such as the number of children they had.

### Number of ANC visits

Our result demonstrating that 70% of the women received four or more ANC visits is comparably higher than the four or more ANC coverages delineated in previous NDHS studies in Nepal: 50% in 2011 [25] and 29% in 2006 [26]. However, recent national-level demographic surveys of low-and middle-income countries such as India (2015), Bangladesh (2014) and Afghanistan (2015) demonstrated the coverage of at least four ANC visits to be 50% [27], 31.3% [28] and 16.5% [29], respectively.

The strongest predictor of the number of ANC visits in our study was the wealth index of a woman’s household. This finding is similar to the study analyzing the 2011 NDHS which found that the women who belonged to the households in the richest wealth quantiles had three times higher the odds of receiving at least four ANC compared to the poorest wealth quantiles [10]. The further analysis of the demographic survey in Bangladesh [28], Afghanistan [29], India [30], and Ethiopia [31] also showed higher odds of four or more ANC visits among women of a higher wealth index compared to those who had lower wealth index. The other predictors that were positively associated with an increase in the number of ANC visits in our study were the educational status of women and their husbands. Joshi and colleagues analyzed data of the NDHS-2011 and found that women who had higher levels of education and women whose husbands had higher educational attainment were more likely to have a greater number of ANC visits [10]. The studies analyzing Ethiopian Demographic and Health Survey (EDHS) [31], and Bangladesh Demographic and Health Survey (BDHS) [28] demonstrated a similar positive association between women’s educational level and four or more ANC visits. This may be due to better health-seeking behavior of women who had higher educational attainment [32–34].

We found that women who had more than two living children were likely to have a lower number of ANC visits compared to those who had at least two living children. Similar findings were observed in a previous study analyzing NDHS-2011 which revealed that for a one unit increase in parity, there were 22% lower odds of receiving four or more ANC visits [10]. Our findings are also consistent with the findings from further analysis of EDHS [31], secondary analysis of the population survey of India [30], and other studies [35, 36]. This may be due to delayed initiation of ANC among women who had greater parity as evidenced by previous systematic reviews [37, 38]. However, an analysis of BDHS [28], and a study in rural and urban surveillance sites of Vietnam [39] showed no association of parity and number of ANC visits.

### Components and quality of ANC

Our study demonstrated that only 21% of Nepalese women received good quality ANC. Although the components determining good quality ANC in the analysis of NDHS 2011 were fewer in our study, our finding is consistent with its finding; it showed that 24% of women received good quality ANC [10]. Similarly, further analysis of the population-based national survey in India showed that less than 25% of women of all ages received adequate ANC [30]. Further, we found that the components of ANC received by the majority of women included the use of two or more doses of tetanus toxoid, measurement of blood pressure, and an ANC visit within the fourth month of pregnancy. In our study, 93.6% of women received two or more doses of tetanus toxoid which is higher than that found in a secondary analysis of NDHS-2011(86.2%); the coverage of all other components of ANC in our study was found to be higher as compared to the previous study [10]. Further, we found that only 11% of women received voluntary HIV counseling which was somewhat higher in the secondary analysis of Nigerian Demographic Health and Household Survey of 2013 (41.7%) [40]. Low coverage of HIV counseling in our study may be due to inadequate training to health workers and lack of a widespread availability of HIV testing materials [41].

Our study revealed that disadvantaged ethnic groups were less likely to have good quality ANC as compared to advantaged ethnic groups. Consistent with our study, socially disadvantaged castes of other groups were less likely to have adequate ANC compared to the advantaged castes in a similar study conducted in India [30]. Such findings may be due to a low level of awareness among disadvantaged ethnic groups; a study in the most disadvantaged ethnic group in Nepal revealed that the women in this group viewed antenatal visits as unnecessary [42], thinking pregnancy as a normal phenomenon that do not require health care visitation. Further, we found that increases in parity were associated with a decreased likelihood of receiving good quality ANC. Similar to our finding, Joshi et al. demonstrated that, in the 2011 NDHS study, with a one unit increase in parity, there were 21% lower odds of receiving good-quality ANC [10]. Our study revealed that literate women and women of higher wealth index were more likely to have good-quality ANC compared to those who are illiterate and women of poor wealth index, respectively. The association of women’s literacy and their socio-economic status and their increased likelihood of receiving good quality ANC was observed in various studies [10, 40, 43]. This could be due to an increased level of awareness and empowerment among educated women.

Our study showed that more than 90% of Nepalese women received two or more doses of tetanus toxoid which signifies that Nepal is on track to sustain the elimination status of neonatal tetanus [44]. Blood pressure measurements of more than 9 in 10 women in our study demonstrate that we can expect a higher chance of early detection of pregnancy-induced hypertension—one of the common causes of maternal mortality in Nepal [45]. According to the National HIV Strategic Plan 2016–2021 [46], Nepal has targeted to reach 90% pregnant women so that they could know their HIV status; however, observing the low coverage of counseling and testing of HIV among pregnant women, this target seems challenging. Further, our finding of 60% of women not receiving at least 180 iron tablets during their pregnancy bolsters the fact of the high prevalence of anemia among pregnant women in Nepal [9]. Since we found a comparatively lower coverage of laboratory services like blood tests and urine tests among various components of antenatal care, establishing laboratory services at the health posts—the main service outlets for ANC service in Nepal [12]— might enhance the quality of ANC. This can be done by the local government as mandated by the recently revised federal health care system of Nepal [47]. Observing our findings, in the short run, targeted interventions such as health education campaigns directed to uneducated women and increasing financial incentives to poor women who seek ANC visits might be helpful. However, in the long run, the overall empowerment of women in terms of education and employment might be needed for sustainable improvement in the frequency and quality of ANC.

### Limitations

Although our study used data from a nationally representative sample of women from a demographic health survey—a methodologically robust survey conducted by trained human resources having a response rate of 98.3% among women aged 15–49— it has some limitations. First, the survey is intrinsically retrospective and thus we could not eliminate recall bias completely. However, we tried to minimize bias by including the information from each woman’s latest ANC visit. Second, the information was self-reported; therefore, response bias including social desirability bias is inevitable. We defined good quality ANC from the perspective of the service received by women; however, we could not measure other characteristics of quality of ANC such as effectiveness, efficiency, equitability, and safety. At the same time, we did not analyze the quality of ANC from the perspective of types of health facilities where the women received ANC.

## Conclusions

Despite a high proportion of four or more ANC visits among Nepalese women, we found that only 21% of women received good-quality ANC. Further, our study revealed that both the frequency and quality of ANC were associated with a breadth of sociodemographic factors. Observing the strong predictors of frequency and quality of ANC such as ethnicity, educational status of women and their husbands, and the wealth index of a woman’s household, Nepal needs targeted interventions directed to disadvantaged, uneducated women and their husbands and economically poor women. Similarly, to enhance the quality of ANC, Nepal needs a greater emphasis on the ANC components where the coverage is low. Our finding of a reduction of the frequency and quality of ANC among women who had a greater number of living children suggests further research to explore the factors behind it. Therefore, a comprehensive approach is highly needed to increase the frequency and enhance the quality of ANC.

## Data Availability

Data can be accessed through website of the DHS program (www.dhsprogram.com).

## Abbreviations

AOR: Adjusted Odds Ratio
APR: Adjusted Prevalence Ratio
ANC: Antenatal Care
FANC: Focused Antenatal Care
NDHS: Nepal Demographic Health Survey
WHO: World Health Organization

## References

[1] WHO | Maternal mortality. *WHO*, http://www.who.int/gho/maternal_health/mortality/maternal_mortality_text/en/ (accessed 15 June 2020).

[2] Maternal mortality. https://www.who.int/news-room/fact-sheets/detail/maternal-mortality (accessed 16 June 2020).

[3] Singh K, Story WT, Moran AC (2016). Assessing the Continuum of Care Pathway for Maternal Health in South Asia and Sub-Saharan Africa. Matern Child Health J.

[4] de Jongh TE, Gurol–Urganci I, Allen E (2016). Integration of antenatal care services with health programmes in low– and middle–income countries: systematic review. J Glob Health.

[5] Berhan Y, Berhan A (2014). Antenatal care as a means of increasing birth in the health facility and reducing maternal mortality: a systematic review. Ethiop J Health Sci.

[6] Tekelab T, Chojenta C, Smith R (2019). The impact of antenatal care on neonatal mortality in sub-Saharan Africa: A systematic review and meta-analysis. PloS One.

[7] World Health Organization, editor. WHO recommendations on antenatal care for a positive pregnancy experience. Geneva: World Health Organization; 2016. 28079998

[8] World Health Organization. Quality of care: a process for making strategic choices in health systems, https://apps.who.int/iris/handle/10665/43470 (2006).

[9] Ministry of Health, New Era, ICF (2017). Nepal demographic and health survey 2016.

[10] Joshi C, Torvaldsen S, Hodgson R (2014). Factors associated with the use and quality of antenatal care in Nepal: a population-based study using the demographic and health survey data. BMC Pregnancy Childbirth.

[11] Ministry of Health and Population. Nepal Health Sector Strategy 2015-2020. Nepal: Kathmandu; 2015. http://www.nhssp.org.np/NHSSP_Archives/health_policy/NHSS_english_book_2015.pdf. Ministry of Health and Population, Nepal.

[12] Lama TP, Chaulagain M, Rai A, et al. *Assessment of the Quality of Antenatal Care, Family Planning, and Sick Child Care Services in Nepal: Further Analysis of the 2015 Nepal Health Facility Survey.* DHS Further Analysis Reports No. 123, Rockville. Maryland: ICF; 2019. https://www.dhsprogram.com/pubs/pdf/FA123/FA123.pdf.

[13] World Health Organization (2002). WHO antenatal care randomized trial: manual for the implementation of the new model. 9241546298, World Health Organization.

[14] National Collaborating Centre for Women’s and Children’s Health (UK. *Antenatal care: routine care for the healthy pregnant woman*. RCOG press, 2008. 21370514

[15] Trujillo JC, Carrillo B, Iglesias WJ (2014). Relationship between professional antenatal care and facility delivery: an assessment of Colombia. Health Policy Plan.

[16] Khatri RB, Mishra SR, Khanal V. Female Community Health Volunteers in Community-Based Health Programs of Nepal: Future Perspective. *Front Public Health*; 5. Epub ahead of print 21 July 2017. DOI: 10.3389/fpubh.2017.00181. 10.3389/fpubh.2017.00181 PMC5519587 28785555

[17] Bloom SS, Lippeveld T, Wypij D (1999). Does antenatal care make a difference to safe delivery? A study in urban Uttar Pradesh, India. Health Policy Plan.

[18] Barber S (2006). Does the Quality of Prenatal Care Matter in Promoting Skilled Institutional Delivery? A Study in Rural Mexico. Matern Child Health J.

[19] Tafere TE, Afework MF, Yalew AW. Providers adherence to essential contents of antenatal care services increases birth weight in Bahir Dar City Administration, north West Ethiopia: a prospective follow up study. *Reprod Health*; 15. Epub ahead of print 29 September 2018. DOI: 10.1186/s12978-018-0610-8. 10.1186/s12978-018-0610-8 PMC6162936 30268132

[20] Ghimire U, Manandhar J, Gautam A, et al. Inequalities in Health Outcomes and Access to Services by Caste/Ethnicity, Province, and Wealth Quintile in Nepal.DHS Further Analysis Reports No. 117. Maryland: ICF: Rockville; 2019.

[21] Bennett L, Dahal DR, Govindasamy P. *Caste, ethnic, and regional identity in Nepal: further analysis of the 2006 Nepal Demographic and Health Survey*. Population Division, Ministry of Health and Population, Government of Nepal, 2008.

[22] Adhikari R (2016). Effect of Women’s autonomy on maternal health service utilization in Nepal: a cross sectional study. BMC Womens Health.

[23] StataCorp L. Stata statistical software: Release 15 (2017). *Coll Stn TX StataCorp LP*.

[24] Datasets for Stata Survey Data Reference Manual. Release 14, https://www.stata-press.com/data/r14/svymain2.html (accessed 21 June 2020).

[25] Ministry of Health and Population (MOHP)[Nepal] NE ICF International Inc. Nepal demographic and health survey 2011.

[26] Nepal. Ministry of Health & Population, NERA (Firm, Kathmandu, et al. *Nepal demographic and health survey, 2006*. Population Division, Ministry of Health and Population, 2007.

[27] IIPS I. National Family Health Survey (NFHS-4), 2015–16. *Int Inst Popul Sci IIPS Mumbai India*.

[28] NIPORT M, ICF. *Bangladesh demographic and health survey 2014*. Technical report, National Institute of Population Research and Training (NIPORT), Mitra and Associates, and ICF International, Dhaka, Bangladesh, and Rockville, Maryland, USA: NIPORT, Mitra and Associates, and ICF International. 2013.,http://microdata. worldbank. org/index. php/catalog/2562; 2016.

[29] Azimi MW, Yamamoto E, Saw YM (2019). Factors associated with antenatal care visits in Afghanistan: secondary analysis of Afghanistan Demographic and Health Survey 2015. Nagoya J Med Sci.

[30] Singh L, Dubey R, Singh S (2019). Measuring quality of antenatal care: a secondary analysis of national survey data from India. BJOG Int J Obstet Gynaecol.

[31] Yaya S, Bishwajit G, Ekholuenetale M (2017). Timing and adequate attendance of antenatal care visits among women in Ethiopia. PLOS ONE.

[32] Lam Y, Broaddus ET, Surkan PJ (2013). Literacy and healthcare-seeking among women with low educational attainment: analysis of cross-sectional data from the 2011 Nepal demographic and health survey. Int J Equity Health.

[33] Mainuddin A, Ara Begum H, Rawal LB (2015). Women Empowerment and Its Relation with Health Seeking Behavior in Bangladesh. J Fam Reprod Health.

[34] Sallis JF, Owen N, Fisher EB, Glanz K, Rimer BK, Viswanath K (2008). Chapter 20: Ecological models of health behavior. Health behavior and health education: theory, research, and practice.

[35] Asweto CO, Aluoch JR, Obonyo CO, et al. Maternal Autonomy, Distance to Health Care Facility and ANC Attendance Findings from Madiany Division of Siaya County, Kenya. Epub ahead of print August 2014. DOI: DOI:10.12691/ajphr-2-4-5.

[36] Fotso JC, Ezeh A, Oronje R (2008). Provision and Use of Maternal Health Services among Urban Poor Women in Kenya: What Do We Know and What Can We Do?. J Urban Health.

[37] Hajizadeh S, Ramezani Tehrani F, Simbar M (2016). Factors Influencing the Use of Prenatal Care: A Systematic Review. J Midwifery Reprod Health.

[38] Feijen-de Jong EI, Jansen DE, Baarveld F (2012). Determinants of late and/or inadequate use of prenatal healthcare in high-income countries: a systematic review. Eur J Public Health.

[39] Tran TK, Gottvall K, Nguyen HD (2012). Factors associated with antenatal care adequacy in rural and urban contexts-results from two health and demographic surveillance sites in Vietnam. BMC Health Serv Res.

[40] Fagbamigbe AF, Idemudia ES (2015). Assessment of quality of antenatal care services in Nigeria: evidence from a population-based survey. Reprod Health.

[41] *Nepal Health Facility Survey* 2015. Kathmandu, Nepal: Ministry of Health, Nepal; New ERA, Nepal; Nepal Health Sector Support Program (NHSSP); and ICF, https://dhsprogram.com/pubs/pdf/SPA24/SPA24.pdf (2017).

[42] Chaurasiya SP, Pravana NK, Khanal V (2019). Two thirds of the most disadvantaged Dalit population of Nepal still do not deliver in health facilities despite impressive success in maternal health. PLOS ONE.

[43] Chandrashekar S, Rao RSP, Nair NS (1998). Socio-Demographic Determinants of Antenatal Care. Trop Doct.

[44] Vandelaer J, Partridge J, Suvedi BK (2009). Process of neonatal tetanus elimination in Nepal. J Public Health.

[45] Suvedi BK, Pradhan A, Barnett S, et al. Nepal maternal mortality and morbidity study 2008/2009: summary of preliminary findings. Kathmandu Nepal Fam Health Div Dep Health Serv Minist Health Gov Nepal.

[46] National HIVSPlan. 2016*–2021*. Kathmandu, Nepal: National Center for AIDS and STD Control, Ministry of Health and Population, https://reliefweb.int/sites/reliefweb.int/files/resources/National%20HIV%20Strategic%20English%202016-2021%20second%20edition_0.pdf (2017).

[47] Thapa R, Bam K, Tiwari P (2018). Implementing Federalism in the Health System of Nepal: Opportunities and Challenges. Int J Health Policy Manag.

